# A Rare Case of Complete Inguinoscrotal Bladder Herniation With Ureteric Involvement: Assessing Diagnostic Challenges and Complex Surgical Management

**DOI:** 10.7759/cureus.55130

**Published:** 2024-02-28

**Authors:** Darcy A Davis, Ronald W Glinski, Matthew C Jones

**Affiliations:** 1 Medicine, Edward Via College of Osteopathic Medicine, Spartanburg, USA; 2 Urology, McLeod Regional Medical Center, Florence, USA; 3 General Surgery/Robotic-Assisted Surgery, McLeod Regional Medical Center, Florence, USA

**Keywords:** ureteric involvement, hydronephrosis, complete bladder herniation, bladder hernia, inguinoscrotal hernia

## Abstract

Inguinoscrotal hernias involving the urinary bladder are exceedingly rare, constituting a small subset of inguinal hernias. We present a case of a 47-year-old male with long-standing scrotal enlargement and obstructive uropathy due to complete herniation of the bladder with ureteric involvement. Diagnostic imaging confirmed the condition. Following an open laparotomy, the bladder was reduced, and a modified Bassini technique with orchiopexy was used for repair. Recurrence of the inguinoscrotal hernia with evidence of the bladder in the scrotal sac required additional surgery. This case underscores the rarity, diagnostic complexity, and potential complications of inguinoscrotal bladder hernias. Specialized surgical techniques and a multidisciplinary approach are crucial for successful management, especially in cases of complete bladder herniation. Future considerations should include innovative approaches to enhance primary repair outcomes for extensive hernias involving the bladder.

## Introduction

Inguinoscrotal hernias of the bladder are exceedingly rare. The involvement of the urinary bladder in inguinal hernias is 1-4%, with complete herniation of the bladder into the scrotum making up a smaller minority of these cases [1]. Levine first described an inguinal bladder hernia as a scrotal cystocele [2]. Following the initial publication, inguinal bladder hernias have been selectively reported in the literature as case reports and case series. Oruç et al. reviewed 190 published cases of inguinal bladder hernias, revealing that only 57 were listed as scrotal hernias with partial bladder involvement in the scrotal sac [1]. Fewer reports have described complete herniation of the bladder into the scrotum [3,4].

Diagnosing an inguinal bladder hernia is often delayed because the presenting symptoms are non-specific and difficult to differentiate from a classical inguinal hernia. The most common symptoms include inguinal swelling followed by lower urinary tract symptoms (LUTS), inguinal pain, and two-stage voiding [5]. Two-stage voiding includes spontaneous urination followed by manual compression of the inguinal swelling with an associated decrease in the scrotum size following micturition [6].

The gold standard for evaluating inguinal bladder herniation is voiding cystography, which will show the characteristic “dog-ear” shaped bladder [6]. However, various imaging types have been reported, including ultrasound and CT [5]. Despite preoperative imaging, most (77%) inguinal bladder hernias are diagnosed intraoperatively [7,8].

Male sex, advanced age, obesity, and weak abdominal muscles are all risk factors for inguinal bladder hernias [9]. The peak incidence of inguinal bladder hernias appears in the sixth decade of life [8,10]. Inguinal bladder hernias are associated with benign prostatic hypertrophy (BPH), bilateral hydronephrosis with or without acute renal failure, bladder stones, vesicoureteral reflux, bladder necrosis, and scrotal abscess [7]. While infrequent, herniation of the ureter into the hernia sac may present with hydronephrosis and renal failure [11]. Genitourinary contents in the scrotal sac lead to complications that necessitate surgical treatment.

Inguinoscrotal hernias of the bladder are treated with manual reduction of the urogenital contents back into the abdomen and repair of the hernia [5]. Open surgical repair has been most frequently reported for managing inguinal bladder hernias; however, the literature has also reported laparoscopic and robotic approaches [4,12]. Khan et al. described two cases using a laparoscopic approach using four ports via Hassan technique to visualize and reduce hernias [4]. Different open techniques described to manage bladder hernias include Lichtenstein, Bassini, McVay, and Shouldice [5]. Bladder injury is the main complication associated with surgical repair of inguinoscrotal hernia of the bladder [5,8].

We report a rare inguinoscrotal hernia with complete bladder herniation, ureteric involvement, and obstruction treated operatively following a modified Bassini technique. The diagnosis was confirmed preoperatively by CT imaging. Although the patient had significant scrotal enlargement and characteristic two-stage voiding for years, the herniation of the bladder was not addressed until hospitalization for a cardiac arrest.

## Case presentation

**Figure 1:**
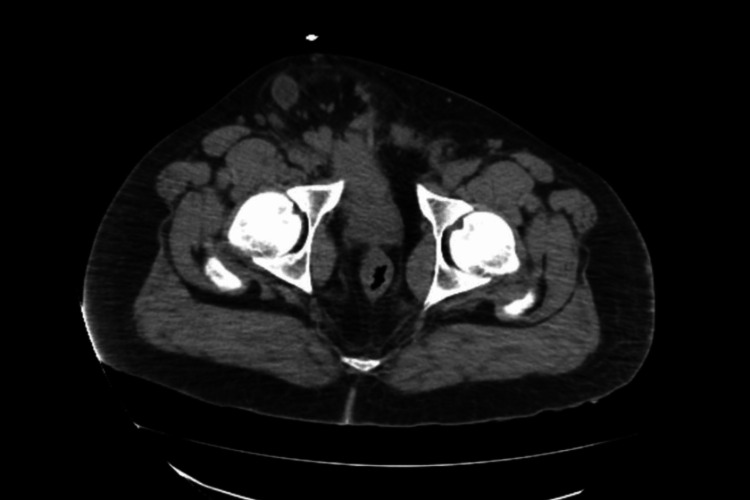
Axial view on CT demonstrating distal ureteric involvement into the scrotum.

**Figure 2:**
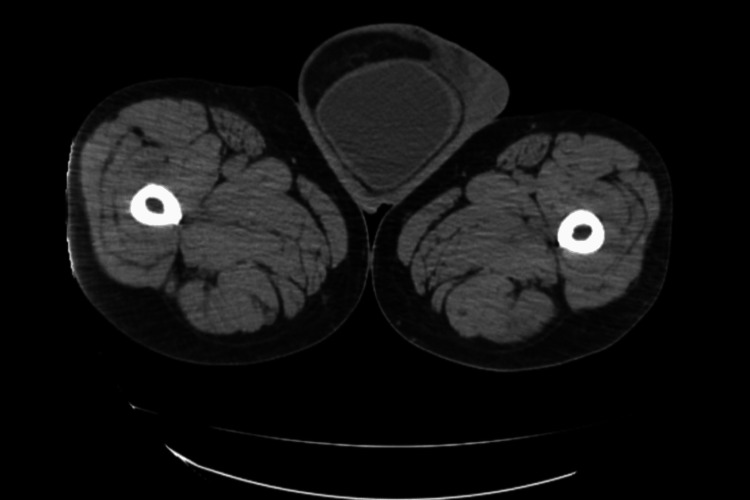
Axial view on CT demonstrating bladder in the right hemiscrotum.

**Figure 3:**
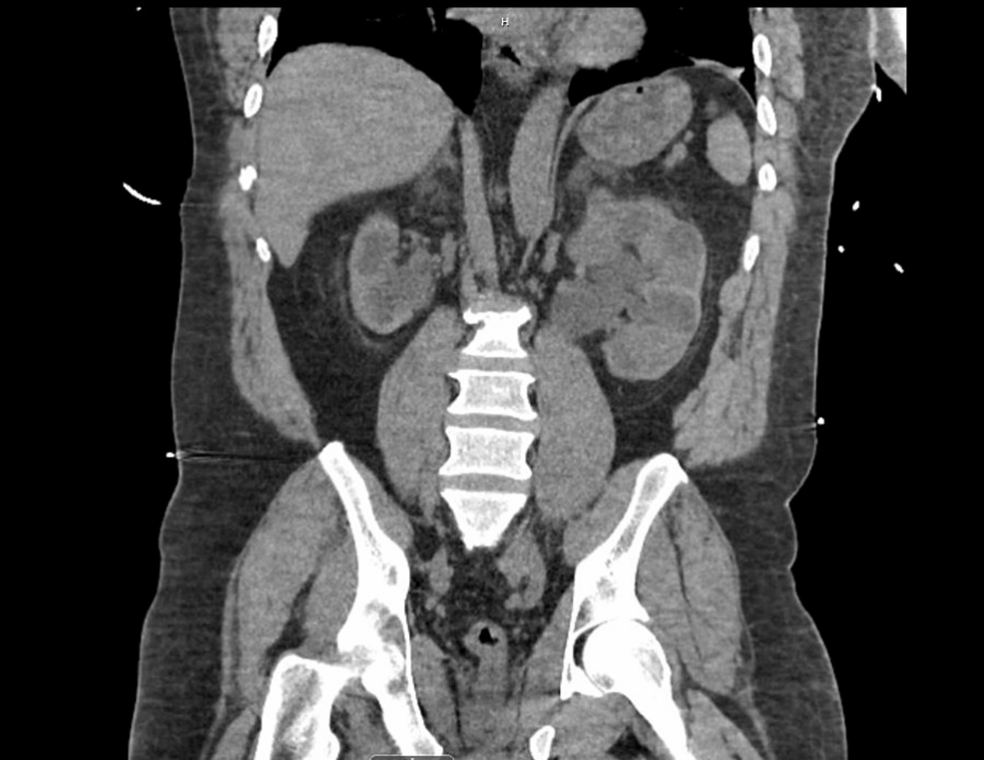
Coronal view on CT demonstrating severe left hydronephrosis with perinephric fat stranding.

A 47-year-old African American male presented to the emergency department after a witnessed cardiac arrest. He was found in pulseless electrical activity, but spontaneous circulation was restored after cardiopulmonary resuscitation. Initial laboratory studies showed elevated creatinine at 5.12, with no known history of renal failure. CT chest/abdomen/pelvis revealed a large right inguinal hernia containing the bladder and distal ureters (Figures 1-2) and severe left hydroureteronephrosis (Figure 3).

The patient reported scrotal swelling for over five years, with minimal discomfort, and he manually decompressed his scrotum to empty the bladder after an initial void. His past medical history included obesity, cocaine abuse, and alcohol abuse. On physical examination, the right hemiscrotum was enlarged without erythema or induration. A Foley catheter was placed, the bladder was drained effectively, and the Foley balloon was noted in the scrotum during the examination.

Two days later, the patient underwent a planned robotic repair of the right inguinal hernia that required conversion to an open laparotomy. The hernia was reduced, revealing a large hernia sac containing the entirety of the bladder. The bladder appeared as if it may have been atonic due to its large size and somewhat “stretched-out” appearance, but it was uninjured. The hernia was repaired with OviTex™ (TELA Bio, Inc., Malvern, USA) mesh following a modified Bassini technique, and orchiopexy was performed.

**Figure 4:**
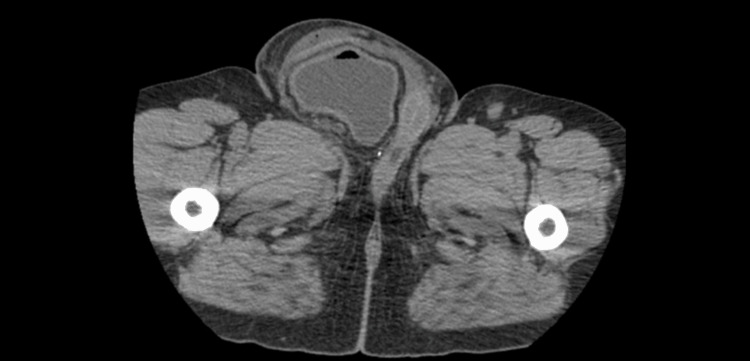
Axial view on CT demonstrating large right groin hernia containing bladder and fat with severe scrotal wall edema.

On postoperative day 6, the patient experienced delayed return of kidney function, and bilateral percutaneous nephrostomy tubes were placed to alleviate severe bilateral hydronephrosis. A follow-up CT scan revealed herniation of the bladder into the right inguinal canal, extending into the right hemiscrotum, with additional severe bilateral hydronephrosis (Figure 4).

The patient returned to the operating room one week later for repeat repair of the defect. Intraoperatively, the leftmost wing of the bladder was adhered to the inferior portion of the laparotomy. The right wing of the bladder had herniated below the previous mesh graft toward the scrotum. Antegrade nephrostograms showed obstructions in both kidneys due to kinking of the ureters and distention. The defect in the right groin was repaired using a peritoneal flap combined with biologic/permanent OviTex™ mesh, anchored into the underlying fascia and muscle. The spermatic cord was ligated due to extensive scar tissue and adhesions. A standard mesh was anchored to the pubic tubercle.

On postoperative day 2, the patient had delayed return of bowel function. Scrotal edema and the right nephrostomy tube output decreased, indicating poor kidney function due to ureteral obstruction. The left nephrostomy had adequate, clear yellow output.

**Figure 5:**
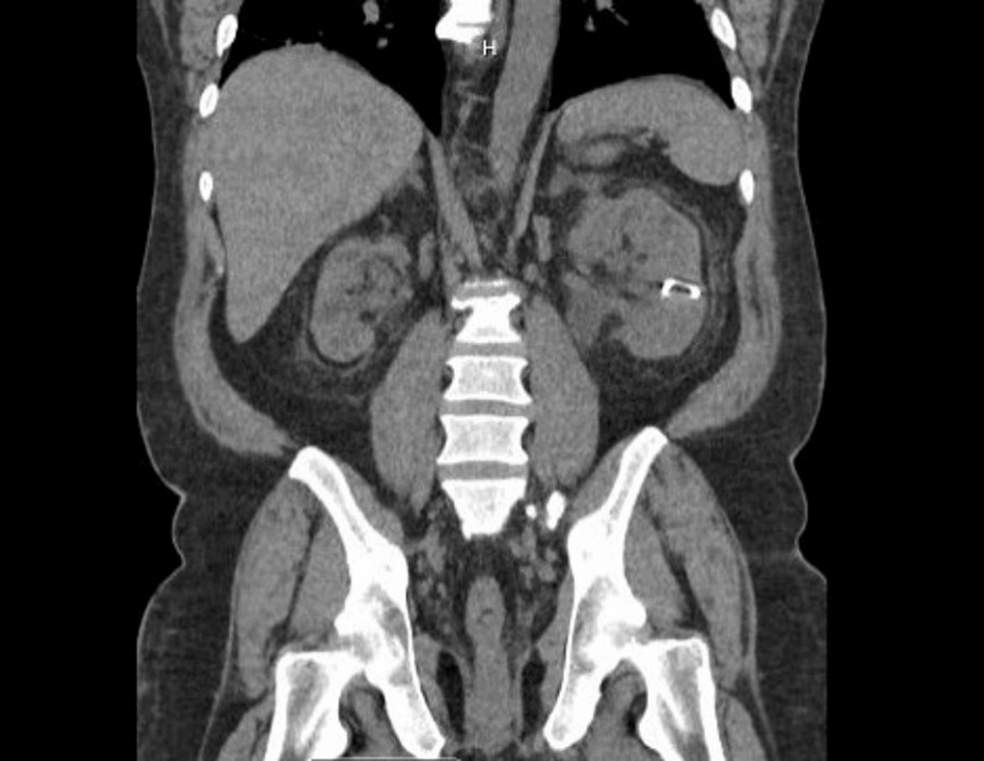
Coronal view on CT showing improved hydronephrosis with residual patulous ureters, with urinary bladder now positioned within the pelvis. The left percutaneous nephrostomy tube appears just outside of the calyx.

At the patient’s one-month follow-up visit with the urologic office, dramatic improvements were noted, with CT imaging showing hydronephrosis resolving (Figure 5). Imaging was consistent with appropriate mesh graft placement, and the bladder was absent from the scrotal sac. The postoperative course was complicated by wound infection, which was resolved with appropriate local therapy.

## Discussion

Inguinoscrotal hernias of the bladder are extremely rare and introduce diagnostic challenges, potential complications, and the need for specialized surgical approaches. Virtually any abdominal or pelvic organ can become trapped in the hernia sac, including the bowel (small, large, and appendix), omentum, bladder, bladder diverticulum, ureter, or lower urinary tract [4,9,13,14]; however, the incidence of genitourinary incarceration is relatively low despite the proximity to the inguinal canal. Inguinoscrotal hernias of the bladder constitute only a small percentage of all inguinal hernias, with complete bladder herniation even more uncommon.

Inguinoscrotal hernias with complete bladder herniation represent a select minority of the published cases in the literature [3,15]. Habib described an inguinoscrotal hernia with complete bladder herniation with complications, including hydronephrosis and acute renal failure that resolved following treatment of the defect [3]. In our case, the patient presented with bilateral hydroureteronephrosis and a large right inguinal hernia containing nearly the entirety of the urinary bladder, distal ureters, sigmoid colon, and distal small bowel. Hydronephrosis and acute renal failure were similarly present in our patient and resolved following the repeat correction of the defect. Adult males with persistent LUTS should be evaluated for signs of obstruction. While the index of suspicion for inguinal bladder herniation is low because of the incidence in the population, all sources of mechanical obstructive uropathy, including inguinal bladder herniation, can increase a patient's risk for severe complications, such as ureteral obstruction, leading to renal injury.

Diagnosing inguinoscrotal hernias of the bladder can be difficult due to their non-specific symptoms and rarity. Clinical suspicion should be heightened when a patient presents with an appreciable groin hernia that reduces following micturition and two-stage voiding symptoms. Inguinoscrotal hernia of the bladder can cause LUTS, such as two-stage voiding, necessitating manual compression of the hernia [8]. Other etiologies, such as BPH, may present with double voiding due to mechanical obstruction by the prostate. BPH may also present concomitantly with inguinal bladder hernias, necessitating a detailed history and physical examination. Our patient's physical examination revealed a large right inguinoscrotal hernia that decompressed with bladder drainage. The Foley balloon was noted in the scrotum, permitting clinical confirmation of the inguinoscrotal hernia of the bladder. The patient had worsening renal function, necessitating surgical repair.

Repairing an inguinoscrotal hernia of the bladder can be challenging, particularly when the bladder is completely involved. Open surgical repair is most frequently reported for managing inguinal bladder hernias [5]. Our patient had a sizeable defect present for more than five years. Due to the chronicity of the presentation, there was heightened concern for bladder atony, leading to chronic urinary retention. Primary repair followed an open surgical approach, which included a modified Bassini technique. Chronic bladder overdistension and flaccidity may complicate initial repair, leading to the potential for recurrence and bladder dysfunction with concomitant renal failure.

Although not done in this case, when addressing large or complete inguinal herniations of the bladder, securing the bladder to the abdominal wall may improve outcomes and prevent downward pressure from the repair on the bladder. A potential approach is utilizing a suprapubic tube with a balloon to affix the bladder to the abdominal wall with cystopexy. Moreover, integrating a suprapubic catheter mitigates the risk of the bladder falling into the repair and potentially causing a recurrence. Also, a suprapubic tube guarantees effective bladder drainage if concurrent poor bladder function is present.

## Conclusions

This case highlights the rarity of large inguinoscrotal hernias involving the urinary bladder. The diagnostic complexity of this condition is evident by the non-specific symptoms at presentation, often leading to delayed diagnosis and complications, including obstructive uropathy and renal failure. Specialized surgical techniques and a multidisciplinary approach are paramount to successful management, especially when dealing with extensive bladder hernias. Open surgical repair, as demonstrated in this case, remains the most frequently cited management for repair. When recurrence of the hernia is likely, securing the bladder to the abdominal wall may enhance the primary repair outcomes in cases of chronic bladder overdistension.

Although not used in this case, a suprapubic tube would be helpful to accomplish this and allow for effective bladder drainage postoperatively in the setting of voiding dysfunction. As publications of large to near-complete inguinoscrotal bladder hernias continue to populate, future considerations should include the development of standardized approaches and guidelines for managing this surgical presentation to improve patient outcomes.
